# District-level maternal and child health index in Uttar Pradesh: a GIS-based analysis using AHP-TOPSIS on NFHS-5 data

**DOI:** 10.1017/S146342362510073X

**Published:** 2026-01-02

**Authors:** Arshad Ahmed, U. Venkatesh, Om Prakash Bera, Ashoo Grover

**Affiliations:** 1 Department of Community & Family Medicine, All India Institute of Medical Sciences (AIIMS), Gorakhpur, UP, India; 2 Global Health Advocacy Incubator (GHAI), Washington, DC, USA; 3 Indian Council of Medical Research, Ansari Nagar, New Delhi, India

**Keywords:** AHP, maternal and child health, multi-criteria decision making, NFHS-5, spatial disparities, TOPSIS, Uttar Pradesh

## Abstract

**Aim:**

This study aimed to develop a transparent district-level Maternal and Child Health (MCH) index for Uttar Pradesh (UP), India, using a hybrid methodological framework integrating Analytic Hierarchy Process (AHP), Technique for Order Preference by Similarity to Ideal Solution (TOPSIS), and Geographic Information Systems (GIS), to support spatially targeted and equitable health planning.

**Background:**

MCH is a key indicator of equity and effectiveness within health systems, directly impacting the wellbeing of mothers and children. Despite global efforts, many low-and middle-income countries continue to face preventable maternal deaths and child illnesses. In Uttar Pradesh (UP), substantial inter–district disparities in MCH outcomes persist but are often masked by state-level averages, hindering targeted policy and resource allocation.

**Methods:**

We applied a hybrid Multi-Criteria Decision-Making (MCDM) framework. AHP was used to assign weights to nine key MCH indicators covering antenatal care, skilled birth attendance, child immunization, and nutrition based on National Family Health Survey (NFHS-5, 2019–21) data across 75 districts. TOPSIS was then employed to rank districts by overall MCH performance. GIS was used to visualize spatial disparities and identify clusters of high and low performance.

**Findings:**

The MCH Index revealed substantial spatial disparities across UP. Districts such as Barabanki, Mahamaya Nagar, and Unnao ranked highest, while eastern UP and Bundelkhand showed lower performance. AHP assigned the highest importance to skilled birth attendance (22%) and antenatal care visits (22%). TOPSIS rankings highlighted gaps in maternal health services in socioeconomically marginalized districts. GIS mapping identified clusters of vulnerability linked to infrastructure and poverty. The AHP-TOPSIS-GIS framework provides a replicable method for sub-state MCH assessment, enabling policymakers to prioritize underserved districts and reduce geographic health outcomes. The findings underscore the need for decentralized, equity-focused strategies tailored to local contexts. Future research should incorporate temporal changes and socio-environmental factors to strengthen planning and monitoring.

## Introduction

Health, education, and income constitute the foundational pillars of human development, with MCH representing a vital barometer of equity and systemic effectiveness particularly in low-and middle-income countries (LMICs) (Koohpayezadeh *et al.*, 2021). Despite sustained international efforts, including the Safe Motherhood Initiative and the Millennium Development Goals (MDGs), an estimated 295,000 women died from pregnancy-related causes in 2017 deaths that were largely preventable and overwhelmingly concentrated in LMICs (Ramazani *et al.*, 2022; Rosa-Mangeret *et al.*, 2022). Beyond underscoring gaps in access to skilled obstetric care, maternal mortality reveals entrenched disparities in health system capacity and geographic access (Kalindi *et al.*, 2023).

India home to nearly one-sixth of the global population has undertaken significant policy reforms in MCH, aligning its national frameworks with international health targets (Madankar *et al.*, 2024). Flagship programs such as Janani Suraksha Yojana (JSY), Pradhan Mantri Matru Vandana Yojana (PMMVY), and Janani Shishu Suraksha Karyakram (JSSK) have measurably improved institutional deliveries and antenatal care coverage (Mali, 2018; MoHFW, 2015). Between April 2005 and August 2009, more than 21 million women accessed JSY benefits, with recent figures indicating support for 28.2 million mothers in just the last three years (Jain, 2010; Srivastava and K. Sureka, 2025). These efforts have contributed to a substantial decline in India’s maternal mortality ratio (MMR), from 437 per 100,000 live births in 1990 to 103 in 2020 marking significant progress, though the country remains above the Sustainable Development Goal (SDG) 3.1 target of fewer than 70 maternal deaths per 100,000 live births by 2030 (Meh *et al.*, 2022; Raina *et al.*, 2023).

However, these aggregate national improvements conceal persistent spatial and socio-economic disparities, particularly in states like UP. Despite comprising nearly 17% of India’s population, UP records a maternal mortality ratio (MMR) of 167 and an infant mortality rate (IMR) of 38 per 1,000 live births both markedly above national averages (NFHS-5, 2021). These disparities stem in part from the uneven implementation of centrally sponsored health schemes, which are administered by individual states within India’s federal governance structure. As a result, maternal health outcomes remain fragmented due to geographic disparities in governance and infrastructure (Ali and Chauhan, 2020; Crear-Perry *et al.*, 2021).

Maternal health service delivery in India is shaped by a bifurcated governance framework outlined in the Seventh Schedule of the Indian Constitution. While the central government through the Ministry of Health and Family Welfare administers apex institutions such as the All India Institute of Medical Sciences (AIIMS), state governments are primarily responsible for operationalizing services at the grassroots level, including Primary Health Centres (PHCs), Community Health Centres (CHCs), and district hospitals (Goala and Bhattacharjee, 2020). This decentralized architecture enables context-specific flexibility but also engenders significant variability in maternal health outcomes across regions. For instance, the efficacy of the JSY, which offers conditional cash transfers to encourage institutional deliveries, varies widely depending on state-level capacities for outreach and quality assurance (Jain, 2010; Neogi, 2023; Venkatesh *et al.*, 2018). Likewise, the Pradhan Mantri Surakshit Matritva Abhiyan (PMSMA) which guarantees comprehensive antenatal check-ups on the 9th of each month remains underutilized in remote and low-performing districts.

However, the implementation and impact of these programs vary widely across districts due to differences in administrative capacity, healthcare infrastructure, and socio-cultural factors. For example, while some districts have achieved high institutional delivery rates under JSY, others still face gaps in access, awareness, or uptake. These disparities underscore the need for district-level assessments to identify localized bottlenecks and guide region-specific health planning.

The utilization of maternal health services in India is shaped by various socio-economic, demographic, and cultural factors. Studies grounded in the Andersen Behavioral Model highlight the influence of maternal education and household wealth on healthcare access (Pandey and Karki, 2014). Despite significant progress, such as 78% institutional deliveries (NFHS-5, 2021), challenges persist in the quality of care. Low compliance with iron-folic acid supplementation, tetanus toxoid coverage, and awareness of pregnancy complications remains prevalent, particularly in rural and low-literacy regions (Mengistu *et al.*, 2023; Tadesse *et al.*, 2021; Ka *et al.*, 2023). Furthermore, unintended pregnancies, accounting for 16.9%, amplify the risks of adverse maternal outcomes (Dehingia *et al.*, 2020).

India’s health surveillance system is hindered by an overreliance on broad state-level metrics that obscure local disparities. Given India’s vast and diverse population, district-level assessments are crucial for identifying specific health challenges and guiding targeted interventions. While composite indices are widely used to synthesize multidimensional data, traditional methods often employ arbitrary weighting, compromising their robustness, transparency, and policy relevance (Lafuente *et al.*, 2022; Pradhan, 2017). A more rigorous and nuanced approach is essential for developing effective, context-sensitive health strategies.

To address methodological complexities, MCDM techniques, particularly AHP and TOPSIS, provide robust solutions. AHP calculates indicator weights by evaluating variability and inter-criteria correlation, ensuring analytical rigor and transparency (Saxena *et al.*, 2022; Ahmed *et al.*, 2024). TOPSIS ranks districts by measuring their proximity to an ideal benchmark while considering their distance from the nadir (S. Sharma *et al.*, 2024). When coupled with GIS, these methods allow for spatial mapping of MCH vulnerabilities, offering a data-driven, targeted approach for equitable policy formulation and resource allocation (Ghosh and Ghosh, 2020).

Despite numerous national surveys and health programs, there remains a clear knowledge gap in district-level assessment of MCH in UP. Existing analyses often rely on aggregated state-level metrics, which obscure local disparities and hinder precise, data-driven planning. This lack of granularity limits the effectiveness of targeted interventions and equitable resource allocation.

With over 240 million residents, UP is India’s most populous and socio-demographically diverse state. Its 75 districts vary significantly in health infrastructure, literacy, caste composition, and poverty levels. A spatially disaggregated analysis is essential to capture this heterogeneity and enable region-specific health strategies that reflect local realities.

This study aims to develop a robust, spatially disaggregated MCH Index for UP, addressing critical gaps in current healthcare assessments. The primary objectives are to construct a composite MCH index using AHP-based objective weighting of key indicators, to rank districts by relative performance using the Technique for Order of Preference by Similarity to Ideal Solution (TOPSIS), and to identify vulnerable spatial clusters using GIS for targeted interventions. By integrating statistical rigor with spatial analysis, this approach shifts from broad aggregates to precise, actionable insights, advancing India’s SDG 3.1 targets and promoting evidence-based, equitable healthcare planning.

## Methods

### Study area

**Figure 1.**
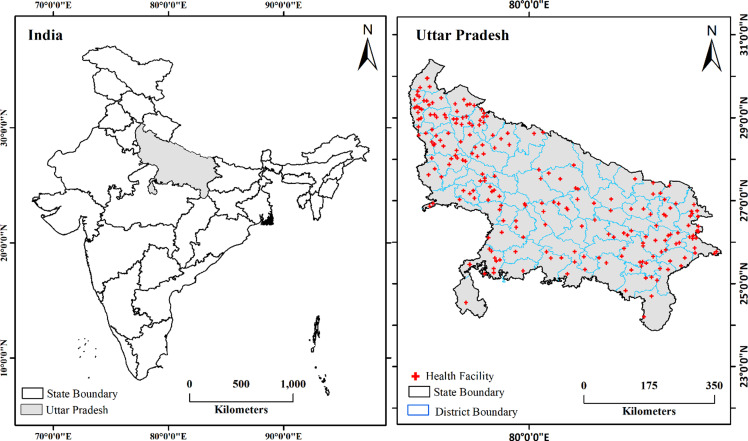
Location of the study area.

Uttar Pradesh is India’s most populous state, comprising 75 districts (Fig. 1). The state’s geographic diversity ranges from the fertile Gangetic plains to hilly and forested terrains, notably in districts like Sonbhadra and Mirzapur. These variations present significant challenges for the delivery of MCH services across districts. Healthcare infrastructure deficits remain pronounced. Rural areas face a 51% shortfall in Community Health Centers (CHCs), and urban regions report a 45% deficit in Primary Health Centers (PHCs) (MoHFW, 2015b). Districts such as Sonbhadra exhibit severe child stunting rates (52.1%) and low ANC coverage (35.8%) (IIPS and ICF, 2021), reflecting the compounded impact of geographic isolation and socioeconomic disadvantage. Conversely, urban districts like Ghaziabad, despite higher institutional delivery rates (83%), report skewed sex ratios (1,182 females per 1,000 males) (NFHS-5, 2021). Socioeconomic vulnerabilities further exacerbate spatial disparities in health inequities. Approximately 32% of UP’s population lives below the poverty line (Arora and Singh, 2025). Regional disparities are especially pronounced in high-risk areas such as Bundelkhand and Purvanchal, where geographic isolation, infrastructural deficits, and seasonal migration patterns hinder timely access to MCH services (Guilmoto, 2023; Yadav, 2024; Yadav *et al.*, 2024). In contrast, districts with stronger NHM infrastructure, such as Meerut, demonstrate improved service coverage, including 92% institutional deliveries (NFHS-5, 2021). UP heterogeneity, encompassing urban centers, tribal regions, and rural agrarian landscapes, provides a critical context for examining spatial disparities across the maternal and newborn CoC.

### Data source

We used data from the fifth round of the NFHS-5, conducted during 2019–2021. This large-scale, nationally representative survey covers all 28 Indian states and 8 union territories, including UP. The survey was conducted by the International Institute for Population Sciences (IIPS), Mumbai, under the guidance of the Ministry of Health and Family Welfare, Government of India. NFHS-5 employs a two-stage stratified sampling design to ensure reliable estimates at national, state/UT, and district levels (IIPS and ICF, 2021).

In the first stage, villages (rural) and Census Enumeration Blocks (urban) were selected using probability proportional to size (PPS), based on the 2011 Census data. PSUs were stratified by rural-urban residence, literacy rates, and socioeconomic characteristics. In the second stage, 22 households per cluster were systematically selected from updated household listings. For UP, the survey covered all 75 districts (as of March 2017), with fieldwork completed in 30,198 out of 30,456 clusters nationally. Detailed sampling methodology and survey protocols are described in the NFHS-5 national report (http://rchiips.org/nfhs/factsheet_NFHS-5.shtml) (NFHS-5, 2021).

### Study participants

The NFHS-5 interviewed 724,115 women aged 15–49 across approximately 637,000 households in India, with a household response rate of 98.1% and a women’s response rate of 97.2%. For our analysis, we used a subset of (*n* = 93,124) women from UP who reported at least one live birth years preceding the survey. This subset ensures relevance to MCH indicators. UP contributed approximately 12.7% of the national sample, reflecting its large population share (NFHS-5, 2021).

### Conceptual framework and multicriteria approach

To develop a composite index for assessing district-level MCH performance, a hybrid Multicriteria Decision Analysis (MCDA) framework integrating the AHP and the TOPSIS was employed. MCDA techniques are increasingly used in complex decision-making contexts for their capacity to synthesize diverse indicators into meaningful rankings (Voogd, 1982; Bernroider and Schmöllerl, 2013; Ishizaka and Siraj, 2018; Kheraj *et al.*, 2023). AHP was applied for weight determination, followed by TOPSIS for ranking spatial units based on proximity to an ideal MCH performance profile.

Following (Ray, 2014), we selected instrumental indicators such as antenatal care, institutional delivery, and immunization coverage to reflect service-level health inputs that are both actionable and consistently available at the district level. Outcome indicators such as maternal mortality and morbidity were excluded due to data limitations and concerns about reliability at finer spatial scales.

### Indicator selection and hierarchical structuring

Nine indicators, derived from NFHS-5 and relevant to MCH, were selected after a rigorous review of policy relevance, empirical validity, and data availability. The indicators span domains such as service coverage (ANC visits, institutional delivery), quality of care (skilled attendance, timely postnatal care), and preventive health (IFA supplementation, tetanus protection). Table 1 summarizes the indicators, codes, descriptions, and derived weights.

**Table 1.** Selected indicators for MCH assessment with AHP-derived weights and descriptions

Code	Indicator	Weight	Description
C1	Mothers with ≥4 ANC visits (%)	0.22	Foundation of safe pregnancy enables early detection and management of complications.
C2	Skilled birth attendance (%)	0.22	Directly reduces risk of maternal and neonatal deaths during delivery.
C3	Children receiving PNC within 2 days (%)	0.12	Critical period for preventing neonatal deaths especially from infections.
C4	PNC to mothers within 2 days (%)	0.12	Helps identify postpartum hemorrhage, infections major causes of maternal mortality.
C5	IFA supplementation ≥100 days (%)	0.08	Essential to prevent maternal anemia and low birth weight widespread issue in India.
C6	IFA supplementation ≥180 days (%)	0.07	Adds more benefit than 100 days, but compliance is harder still important.
C7	Births protected against neonatal tetanus (%)	0.07	Very important, but high national coverage already reduces urgency.
C8	Institutional births (%)	0.05	Has improved significantly in India less variability across regions now.
C9	MCP card received (%)	0.05	Important for record-keeping and continuity of care, but indirect impact on outcomes.

The hierarchical structure underpinning the AHP model consists of three levels:

1. Goal: Assessing and ranking district-level MCH performance in UP.
2. Criteria: Nine MCH indicators (C1–C9).
3. Alternatives: The 75 districts of UP.

Although MCDM methods have traditionally been used in engineering and finance, their application in healthcare has expanded significantly, particularly for complex MCDM scenarios such as resource allocation, service prioritization, and policy evaluation (Taherdoost and Madanchian, 2023). In MCH, where interventions span service coverage (e.g., ANC visits), quality (e.g., skilled birth attendance), and preventive care (e.g., immunization), MCDM provides a structured framework to reconcile competing priorities and spatial inequities. Recent studies have demonstrated its utility in health system planning, including hospital location selection (Ghosh and Ghosh, 2020) and regional health vulnerability assessments (Saxena *et al.*, 2022). Given UP’s heterogeneous MCH landscape, the AHP-TOPSIS-GIS hybrid approach enables transparent, data-driven prioritization of districts for targeted interventions.

### Weight derivation using the AHP

Pairwise comparisons of indicators were conducted by the authors based on a structured review of national health program guidelines, existing public health literature, and indicator relevance in the Indian context. Although a formal expert panel was not convened, the judgments reflect informed prioritization aligned with key MCH programmatic focus areas. This approach is consistent with established practices in healthcare-related AHP applications, particularly when using structured literature-based judgments in the absence of expert consensus panels (Schmidt *et al.*, 2015).

The Saaty 1–9 scale was applied (Saaty, 2008), and the resulting reciprocal matrix was normalized. Relative weights were computed by averaging across normalized rows. Consistency was evaluated through the Consistency Index (CI) and Consistency Ratio (CR), where:

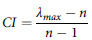




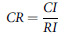

where 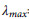 =9.474, *n* = 9, and the standard Random Index (RI), the computed CR was 0.041, indicating acceptable consistency (*CR* < 0.1), thereby validating the reliability of the weight matrix. Higher weights were accorded to indicators reflecting service access and skilled care, such as ANC visits (C1) and skilled birth attendance (C2).

### Data normalization

To ensure comparability across indicators with differing scales and units, min-max normalization was performed. The formulae are:

For beneficial indicators (higher is better):

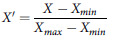




For benefit indicators (lower is better):

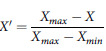




This standardization process yielded dimensionless values ranging between 0 and 1 for all indicators.

### Composite index construction using TOPSIS

Following AHP-derived weighting, the TOPSIS methodology was applied to synthesize a composite score for each district based on the following steps:

1. *Decision matrix formation:* A matrix 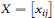 was constructed, where 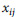 denotes the normalized score for the 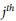 indicator in the 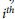 district.
2. ***Weighted normalized matrix:*** Each element of the decision matrix was multiplied by its corresponding AHP-derived weight:

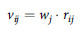

3. *Ideal and the negative-ideal solutions:* The ideal solution 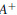 and the negative-ideal solution 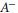 were determined to represent the best and worst performance levels across all criteria, respectively. For health susceptibility assessment in this study, each criterion was considered as a benefit criterion (lower values indicate reduced vulnerability). Thus, the ideal and anti-ideal solutions were calculated as follows:

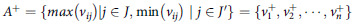




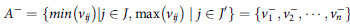

where; 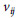 is the weighted normalized value for criterion 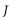 of spatial unit *i*, and 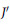 represents the set of benefit criteria in this context (all criteria are treated as benefit criteria due to the focus on reducing vulnerability).
4. *Calculation of the Euclidean distances:* The distances from the ideal solution 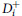 and negative-ideal solution 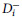 for each spatial unit were computed as:

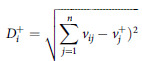




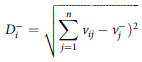

where; 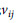 is the weighted normalized value of criterion 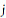 for spatial unit *i*, and 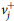 is the value of the ideal solution for criterion 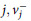 the value of the negative–ideal solution for criterion, and 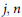 the total number of criteria.
5. *Relative closeness to the ideal solution:* Each spatial unit relative closeness 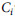 to the ideal solution was calculated using:

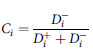

  Where; 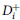 is the Euclidean distance from the ideal solution, 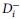 the Euclidean distance from the negative–ideal solution. The closeness coefficient 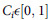 signifies the degree to which a district approximates the ideal MCH performance profile. Higher values indicate better relative performance.
6. *Ranking of districts*: Districts were ranked based on their 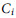 values using the descending order function in Microsoft Excel:

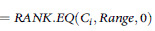

  The third argument, 0, indicates descending order, meaning higher scores receive a better (lower) rank. Rank 1 denotes the best-performing district.

While TOPSIS is commonly used to identify optimal alternatives in decision-making scenarios, its applicability extends to comparative performance analysis across multiple units. In this study, our aim was not to select a single best-performing district but to evaluate and rank all 75 districts based on their proximity to an ideal MCH performance profile. TOPSIS provides a transparent and structured framework to quantify relative closeness to an ideal solution, which helps policymakers identify performance gaps and areas for targeted improvement. Similar applications have been documented in public health and social development settings where ranking and prioritization—not selection are the core objectives. Therefore, the method is well-aligned with the study’s goal of enabling district-level comparison for prioritizing districts in healthcare planning.

### Spatial classification and mapping

To identify spatially targeted health intervention, districts were classified into five performance categories using the Natural Breaks (Jenks) classification method in QGIS (Brewer and Pickle, 2002). This method identifies natural groupings by minimizing within-class variance and maximizing between-class variance. The classification is as follows: Very High Performance (Rank 1–15), High Performance (Rank 16–30), Moderate Performance (Rank 31–45), Low Performance (Rank 46–60), and Very Low Performance (Rank 61–75). These are the most susceptible districts in terms of MCH and should be prioritized for urgent and intensive health interventions. This spatial categorization enables data-driven health planning by clearly delineating areas needing urgent, moderate, or minimal attention, thereby facilitating evidence-based and efficient allocation of healthcare resources across UP.

## Results

### AHP-based weight assignment for MCH indicators

The AHP was applied to assign relative weights to nine key MCH indicators using pairwise comparisons informed by policy relevance and expert judgment. The consistency ratio (CR = 0.041) confirmed acceptable logical consistency (CR < 0.1). Higher weights were assigned to maternal service-related indicators such as antenatal care visits and skilled birth attendance, underscoring their central role in MCH performance. Detailed weighted derivation and consistency matrices are presented in Appendix Tables A1–A3.

### Spatial patterns in MCH indicators

**Figure 2.**
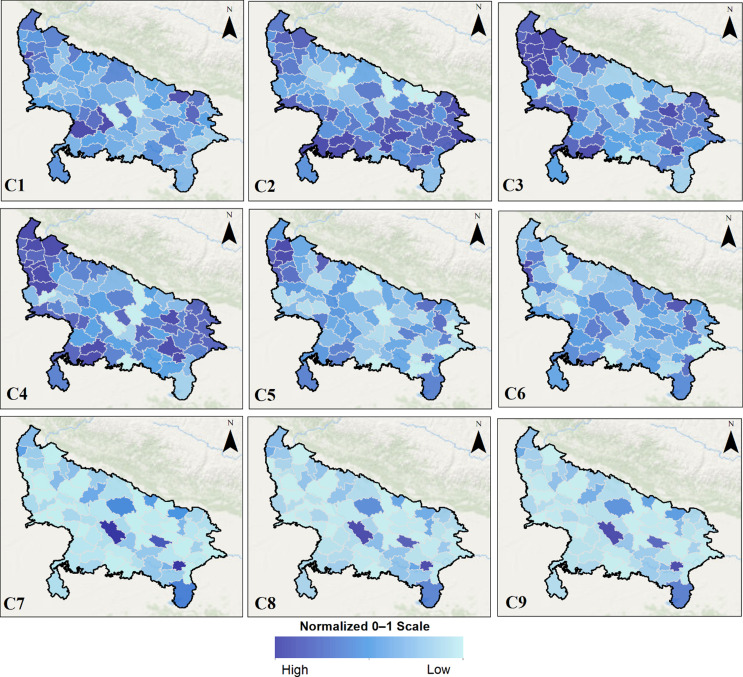
District-wise spatial patterns in nine MCH Indicators (C1–C9) across UP, with darker shades indicating better performance.

Figure 2 illustrates spatial disparities in MCH performance across 75 districts of UP, based on nine weighted indicators (C1–C9) using the TOPSIS framework. These indicators represent key dimensions of MCH, including service coverage, quality of care, nutrition, and access to primary health services.

The analysis reveals clear regional disparities in C1; maternal health service coverage is comparatively lower in eastern districts such as Ballia and Ghazipur, whereas urbanized districts like Gautam Buddha Nagar and Kanpur Nagar show more robust service outreach. C2 highlights significant Skilled birth attendance gaps are observed in Shrawasti and Sitapur, suggesting a need for strengthened immunization drives. C3 and C4 indicate suboptimal performance in institutional deliveries and skilled birth attendance in Bundelkhand and parts of Purvanchal (e.g., Banda, Mahoba, Bahraich), pointing toward systemic gaps in maternity care infrastructure.

In terms of C5 and C6 (ANC and PNC services), districts such as Rae Bareli, Mirzapur, and Sonbhadra lag behind, reflecting uneven distribution of maternal care services, especially in semi-urban or topographically challenging areas. C7 (child undernutrition) draws attention to critical health and nutrition deficits in Sitapur, Sonbhadra, and Varanasi, indicating multifaceted challenges in early childhood care and dietary adequacy.

C8 shows high infant mortality in parts of Eastern UP, underscoring regional gaps in neonatal care. C9 (access to primary health facilities) highlights uneven distribution of service in Unnao and Varanasi, where health infrastructure is disproportionately concentrated or underutilized.

Collectively, the spatial analysis identifies specific district-level performance gaps across MCH indicators. Regions such as eastern UP and Bundelkhand consistently underperforming in multiple health dimensions, reinforcing the need for geographically targeted interventions and integrated maternal-child health planning at the sub-state level.

**Figure 3.**
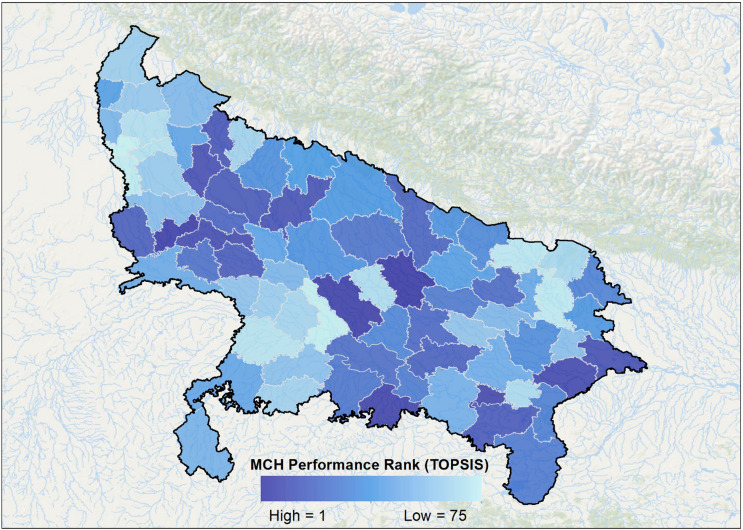
Composite MCH performance rankings of UP districts, classified into five tiers (Very High to Very Low) based on TOPSIS analysis.

The MCH Index developed using a combination of AHP for objective indicator weighting and TOPSIS for composite ranking provides a robust assessment of district-level performance in UP. The final rankings reflect how close each district is to the ideal scenario of optimal MCH outcomes (Figure 3).

Districts such as Barabanki, Mahamaya Nagar, and Unnao emerge as the top performers, suggesting relatively better service coverage, antenatal care, and institutional delivery utilization. These districts can serve as benchmarks for others in terms of policy replication and implementation best practices. On the other hand, Gautam Buddha Nagar, Kanpur Nagar, and Ghaziabad rank lowest, indicating substantial gaps in MCH indicators, likely driven by service inaccessibility, low awareness, or infrastructural deficits (Table 2). These regions merit immediate attention for targeted interventions and resource prioritization. Overall, the results display a wide inter-district disparity, validating the need for spatially targeted, data-driven policymaking rather than blanket statewide strategies. The index facilitates a move away from uniform program design, empowering local governance units to address district-specific vulnerabilities.

**Table 2.** District performance based on TOPSIS rankings

Category	Rank range	District count	Districts
Very High Performance	1–15	15	Barabanki, Mahamaya Nagar, Unnao
High Performance	16–30	15	Pratapgarh, Bahraich, Kanshiram Nagar
Moderate Performance	31–45	15	Balrampur, Azamgarh, Bareilly
Low Performance	46–60	15	Agra, Jaunpur, Prayagraj
Very Low Performance	61–75	15	Gautam Buddha Nagar, Kanpur Nagar, Ghaziabad

This categorization not only highlights zones of excellence and concern but also supports decision-makers in prioritizing policy, funding, and technical interventions tailored to district-specific MCH profiles.

## Discussion

This study presents a comprehensive evaluation of MCH performance across the 75 districts of UP using a hybrid MCDM approach, combining the AHP and the TOPSIS, alongside spatial visualization through GIS. The resulting MCH Index reveals considerable spatial disparities in healthcare access and outcomes, underscoring the need for geographically tailored health policies (Kalindi *et al.*, 2023; Meh *et al.*, 2022).

The AHP-derived weights reflect the prioritization of key indicators such as antenatal care (ANC), institutional deliveries, immunization coverage, nutritional status, and iron-folic acid (IFA) consumption variables that strongly influence maternal and neonatal health outcomes (Mengistu *et al.*, 2023; Ramazani *et al.*, 2022). This weighted framework offers a transparent, data-driven basis for district-wise comparison and resource allocation. The application of TOPSIS complements this by ranking districts against an ideal benchmark, ensuring dynamic and interpretable policy guidance (Saxena *et al.*, 2022; S. Sharma *et al.*, 2024).

The analysis reveals a clear pattern of regional disparities in MCH outcomes. Districts such as Barabanki, Mahamaya Nagar, and Unnao emerge as high performers on the MCHI. These areas demonstrate relatively strong uptake of ANC services, skilled birth attendance, and full immunization coverage. Their improved performance may be attributed to better-functioning health infrastructure, higher levels of female literacy, and effective implementation of programs such as JSY and POSHAN Abhiyaan (MoHFW, 2015a; Mali, 2018; Jain, 2010). The correlation between maternal education and healthcare service utilization in these districts mirrors broader national findings observed in high-performing states like Kerala and Puducherry (Paul and Chouhan, 2020) and aligns with findings from the Andersen Behavioral Model, which emphasizes the role of education and wealth in shaping access to care (Pandey and Karki, 2014).

Conversely, districts like Gautam Buddha Nagar, Kanpur Nagar, and Ghaziabad rank among the lowest. Despite partial urbanization, these regions suffer from low institutional delivery rates, incomplete immunization, and inadequate postnatal care (IIPS and ICF, 2021). Although these are highly urbanized districts, their poor results likely reflect intra‑urban disparities, especially among slum and peri‑urban populations, which are often masked in district-level averages. For example, studies in Kanpur’s slums report high rates of child undernutrition and poor maternal health service use (Rathore *et al.*, 2023). In Varanasi and Unnao, intra-urban inequalities particularly within slum populations contribute to service underutilization. Sonbhadra’s challenges stem from its remote, tribal-dominated geography, echoing healthcare access issues prevalent in hilly and forested terrains such as those in the northeastern states, and reflect underperformance in decentralized service delivery under India’s federal governance structure (Ali and Chauhan, 2020; Goala and Bhattacharjee, 2020). These patterns align with findings by Singh and Chaturvedi (2015), whose study of Empowered Action Groups states (including Uttar Pradesh) documented systemic disparities in ANC coverage affecting marginalized communities.

Furthermore, the spatial clustering of poor MCHI scores in Eastern UP and Bundelkhand points to deeper systemic issues. Districts like Sitapur and Banda consistently show deficiencies in IFA intake, ANC registration, and child nutrition indicators that prior studies link to poverty and caste-based exclusions (Khan *et al.*, 2014; Gupta *et al.*, 2024) as well as low awareness of pregnancy complications and poor quality of antenatal care (Dehingia *et al.*, 2020; Tadesse *et al.*, 2021). These findings align with (Dehury *et al.*, 2017), who emphasized the predictive power of nutritional and maternal care indicators in under-resourced settings.

While our index does not directly measure socioeconomic factors, the clustering of low-performing districts in regions like Bundelkhand which prior studies link to poverty highlights the need for spatially prioritized (Yadav, 2024).

Persistent underperformance in certain districts may partly reflect underlying health system readiness issues, such as limited facility capacity or workforce shortages, which constrain effective service delivery despite existing programs.

The middle-ranked districts, including Balrampur, Azamgarh, and Bareilly, demonstrate mixed performance. Although these districts benefit from better healthcare facilities, disparities within urban poor populations and slum settlements dilute overall MCHI scores. This underscores that even well-equipped districts require micro-targeted interventions to address intra-district disparities (Crear-Perry *et al.*, 2021).

The spatial visualizations derived from GIS mapping further amplify these findings, offering an intuitive tool for policymakers to identify lagging regions and plan geographically specific responses. The granularity provided by MCHI mapping allows for evidence-based microplanning, which is crucial in a large and diverse state like UP (Ghosh and Ghosh, 2020).

From a policy standpoint, the study reinforces the need for decentralized and district-sensitive approaches. In low-ranked districts, interventions should prioritize mobile healthcare units for remote populations, enhanced community health worker outreach, and awareness campaigns on maternal nutrition and postnatal care. Governance mechanisms such as real-time monitoring, inter-sectoral convergence, and capacity building at the primary health center (PHC) level are also essential to bridge service delivery gaps (Neogi, 2023).

Moreover, the methodological framework employed anchored in AHP-TOPSIS and geospatial analysis is replicable and adaptable, offering a scalable model for other low-performing states. It supports prior research advocating for localized health assessments to guide interventions in socio-economically vulnerable regions (Srivastava and K. Sureka, 2025; Goala *et al.*, 2024) and addresses critiques of traditional indices that rely on arbitrary weights (Lafuente *et al.*, 2022; Pradhan, 2017).

Despite UP’s complex health landscape and resource constraints, the success of several districts suggests that targeted strategies, when coupled with community participation and institutional support, can yield measurable improvements. A multi-stakeholder model involving government agencies, local organizations, and community leaders is imperative for achieving long-term impact (Madankar *et al.*, 2024; Raina *et al.*, 2023).

Compared to conventional approaches like equal weighting or PCA, the AHP-TOPSIS-GIS framework offers superior transparency, indicator interpretability, and spatial relevance, making it especially suitable for localized health planning in complex demographic settings like UP.

These findings have direct policy implications. Low-performing districts, especially in Eastern UP and Bundelkhand, should be prioritized for targeted investments in maternal nutrition, postnatal care, and health infrastructure. The composite MCH Index developed here can serve as an evidence-based planning tool to allocate resources, identify service gaps, and design district-specific interventions. Furthermore, integrating this framework with real-time data platforms such as the Health Management Information System (HMIS) could enhance monitoring, enable timely response to emerging health trends, and support adaptive program management.

## Limitations

While this study offers a robust framework for assessing MCH performance in UP using a hybrid AHP-TOPSIS model and GIS-based visualization, several limitations warrant consideration.

First, the analysis relies entirely on secondary data from the National Family Health Survey (NFHS-5), which–although comprehensive–may include self-reported inaccuracies and time lags that affect its real-time relevance. Spatial disaggregation at the district scale, though valuable for planning, can obscure inequalities within larger or more diverse geographic units.

Second, the selection of indicators was shaped by data availability. Key variables such as maternal morbidity, birth spacing, out-of-pocket expenditure, and care quality could not be incorporated due to lack of reliable data. Moreover, structural determinants like caste-based exclusion, women’s decision-making power, and social capital known to influence healthcare access could not be quantified in this study.

Third, although the AHP-TOPSIS approach supports systematic comparison, the weight assignment process is inherently subjective. Moreover, the model assumes static relationships and does not account for dynamic feedbacks or policy shifts over time.

Finally, while GIS mapping spatial enhances spatial understanding, the analysis reflects a single time point and does not capture or seasonal variation in service delivery or disease burden. Future research should explore longitudinal data, real-time health information systems, and community-level primary surveys would provide a more granular and dynamic understanding of MCH disparities.

## Conclusion

This study developed a spatially disaggregated MCH Index for UP using a hybrid AHP-TOPSIS framework integrated with GIS. The analysis revealed significant intra-state disparities, particularly concentrated in Eastern UP and Bundelkhand, emphasizing the need for geographically targeted, equity-oriented health interventions. By identifying multidimensional gaps across districts, this approach offers a replicable model for localized policy planning, performance monitoring, and efficient resource allocation. The findings call for integrated strategies that go beyond spatial diagnostics focusing instead on strengthening community-based healthcare delivery, intersectoral coordination, and culturally sensitive program design for underserved populations.

## Data Availability

The dataset analyzed during the current study are available in the Demographic and Health Surveys (DHS), https://dhsprogram.com/data/available-datasets.cfm.

## Appendix A

**Table A1.** Pairwise comparison matrix creation

Criteria	C1	C2	C3	C4	C5	C6	C7	C8	C9
C1	1	1	2	2	3	3	3	4	4
C2	1	1	2	2	3	3	3	4	4
C3	0.5	0.5	1	1	2	2	2	3	3
C4	0.5	0.5	1	1	2	2	2	3	3
C5	0.333	0.333	0.5	0.5	1	2	2	2	2
C6	0.333	0.333	0.5	0.5	0.5	1	2	2	2
C7	0.333	0.333	0.5	0.5	0.5	0.5	1	2	2
C8	0.25	0.25	0.333	0.333	0.5	0.5	0.5	1	2
C9	0.25	0.25	0.333	0.333	0.5	0.5	0.5	0.5	1
Total	4.50	4.50	8.17	8.17	13.00	14.50	16.00	21.50	23.00

**Table A2.** Utilizing a standardized matrix and weighted distribution in the case of multi-criteria decision analysis

Criteria	C1	C2	C3	C4	C5	C6	C7	C8	C9	Weight
C1	0.22	0.22	0.24	0.24	0.23	0.21	0.19	0.19	0.17	0.22
C2	0.22	0.22	0.24	0.24	0.23	0.21	0.19	0.19	0.17	0.22
C3	0.11	0.11	0.12	0.12	0.15	0.14	0.13	0.14	0.13	0.12
C4	0.11	0.11	0.12	0.12	0.15	0.14	0.13	0.14	0.13	0.12
C5	0.07	0.07	0.06	0.06	0.08	0.14	0.13	0.09	0.09	0.08
C6	0.07	0.07	0.06	0.06	0.04	0.07	0.13	0.09	0.09	0.07
C7	0.07	0.07	0.06	0.06	0.04	0.03	0.06	0.09	0.09	0.07
C8	0.06	0.06	0.04	0.04	0.04	0.03	0.03	0.05	0.09	0.05
C9	0.06	0.06	0.04	0.04	0.04	0.03	0.03	0.02	0.04	0.05
Total	1	1	1	1	1	1	1	1	1	1

**Table A3.** Estimation of the consistency ratio

Criteria	C1	C2	C3	C4	C5	C6	C7	C8	C9	Total	CR
C1	0.21	0.21	0.26	0.26	0.27	0.24	0.21	0.20	0.16	2.020	9.111
C2	0.21	0.21	0.26	0.26	0.27	0.24	0.21	0.20	0.16	2.020	9.111
C3	0.11	0.11	0.13	0.13	0.18	0.16	0.14	0.15	0.12	1.220	9.930
C4	0.11	0.11	0.13	0.13	0.18	0.16	0.14	0.15	0.12	1.220	9.930
C5	0.07	0.07	0.07	0.07	0.09	0.16	0.14	0.10	0.08	0.840	10.421
C6	0.07	0.07	0.07	0.07	0.05	0.08	0.14	0.10	0.08	0.715	9.931
C7	0.07	0.07	0.07	0.07	0.05	0.04	0.07	0.10	0.08	0.605	9.263
C8	0.05	0.05	0.04	0.04	0.05	0.04	0.04	0.05	0.08	0.442	9.214
C9	0.05	0.05	0.04	0.04	0.05	0.04	0.04	0.03	0.04	0.377	8.358
										Total	9.474

Maximum eigenvalue (*λ*_max) = 9.474, *n* = 9; Consistency index (CI) = ((*λ*_max-*n*)/(*n* - 1)) = 0.059; Random index (RI) = 1.45; Consistency ratio (CR) = (CI/RI) = 0.041.
